# High serum levels of transforming growth factor β1 are associated with increased cortical thickness in cingulate and right frontal areas in healthy subjects

**DOI:** 10.1186/1742-2094-9-42

**Published:** 2012-02-28

**Authors:** Fabrizio Piras, Francesca Salani, Paola Bossù, Carlo Caltagirone, Gianfranco Spalletta

**Affiliations:** 1 Fondazione IRCCS Santa Lucia, via Ardeatina 306, 00179 Rome, Italy; 2 Dipartimento di Neuroscienze, Università Tor Vergata, via Montpellier 1, 00133 Rome, Italy

**Keywords:** TGF-β, Cortical thickness, Inflammation, Neuroprotection

## Abstract

**Background:**

Transforming growth factor β (TGF-β) is a cytokine having multiple functions in the central nervous system such as promoting repair mechanisms in degenerative diseases and stroke. To date, however, its neuroprotective effects in non-disease conditions have not been studied

**Methods:**

With the aim of exploring the relationship between peripheral TGF-β1 expression and brain structural integrity, 70 healthy participants underwent high-resolution structural T1-weighted magnetic resonance imaging scans and blood sampling. Data were processed to obtain brain cortical thickness and serum concentrations of TGF-β1. We investigated the correlation between TGF-β1 and cortical thickness using both region-of-interest- and vertex-based approaches.

**Findings:**

Region-of-interest-based analysis of the cortical mantle showed a correlation between TGF-β1 serum concentrations and cortical thickness bilaterally in cingulate and right frontal and temporal areas. Similar results emerged in the vertex-based analysis, where significant correlations were found bilaterally in cingulate and right frontal cortices.

**Conclusions:**

These results suggest that TGF-β1, through its role in down-regulating inflammatory processes, might have a beneficial effect on the structural integrity of the brain in physiological states.

## Background

In mammals, transforming growth factor β (TGF-β) is a multifunctional cytokine which exists as three closely related isoforms (TGF-β1, TGF-β2 and TGF-β3) that bind to the same receptors and exert similar functions but with different spatiotemporal control of their expression patterns [1].

Interestingly, TGF-β effects are prominent in brain development [2] and its signaling might control the size of a specific brain area by modulating self-renewal of neural stem cells [3]. In addition, an ongoing and potent trophic role for this anti-inflammatory cytokine has been identified in TGF-β1-deficient unlesioned as well as injured adult brain [4].

TGF-β is also involved in down-regulating inflammatory reactions to injury and in promoting repair mechanisms [5], and has been implicated in the pathophysiology of chronic neurodegenerative disorders and stroke [6]. Indeed, it has been shown that a reduction of TGF-β signaling increases amyloid deposition and degeneration in transgenic Alzheimer's Disease (AD) mice [7], although negative effects of TGF-β in neurodegeneration have also been described [8,9]. In the vasculature, TGF-β modulates atherosclerosis and restenosis [10]. There is also a great deal of evidence, primarily from animal studies, that TGF-β plays a crucial protective role in reducing infarct size following cerebral ischemia [11,12].

Although TGF-β has been recognized as a neuroprotective factor, the mechanisms underlying the protective effects have yet to be clarified. In vivo evidence for a beneficial role of TGF-β in human brain is scarce or limited to pathological conditions such as ischemia and neurodegenerative diseases, and the pattern of such protection in physiological conditions has not yet been thoroughly investigated. It could be that individual TGF-β phenotypes exert different levels of protection from brain pathologies such as neurodegenerative diseases, vascular deficits and aging.

Following this line of reasoning, the hypothesis of a relationship between TGF-β expression and brain structural integrity would not be unjustified. Thus, the aim of the present pilot study was to determine whether there is an association between peripheral TGF-β1 expression (i.e. concentrations in serum samples) and structural integrity of the brain (i.e. cortical thickness) in healthy human subjects.

## Methods

### Subjects

Seventy healthy subjects (27 males, 43 females; mean age ± sd = 31 ± 8.5 years, range 18-48; mean education ± sd = 15.6 ± 2.8 years, range 8-18) were recruited from universities, community recreational centres and hospital personnel by local advertisement. The inclusion criteria were age between 18 and 50 years and suitability for MRI scanning. Exclusion criteria included: i) suspicion of cognitive impairment or dementia based on a Mini Mental State Examination (MMSE) [13] score ≤ 24, and confirmed by clinical neuropsychological evaluation using the Mental Deterioration Battery [14] and NINCDS-ADRDA diagnostic criteria for dementia [15], ii) subjective complaints of memory difficulties or of any other cognitive deficits, iii) major medical illnesses, iv) current or reported psychiatric or neurological disorders, v) known or suspected history of alcoholism or drug dependence, vi) MRI evidence of focal parenchymal abnormalities or cerebrovascular diseases, and vii) presence of systemic inflammatory diseases and/or treatment with anti-inflammatory drugs at the time of the assessment.

The study was approved and undertaken in accordance with the guidance of our local Ethics Committee and written consent was obtained from all participants.

### TGF-β measurement

Peripheral blood samples were obtained in the early morning from all subjects by venipuncture of an upper limb. Serum TGF-β1 levels were measured by a quantitative enzyme immunoassay (ELISA) technique using a specific TGF-β1 kit (Human TGF-β CytoSet, Biosource, Camarillo, CA, USA) according to the manufacturer's instructions. The calibrator consisted of recombinant human TGF-β1. All samples were measured in duplicate and respective mean values were calculated. The limit detection of the assay was 30 pg/ml and the intra- and inter-assay coefficients of variability were 2.8% and 12.5%, respectively.

### MRI acquisition and cortical thickness analysis

All 70 participants underwent the same imaging protocol, which included standard clinical sequences (FLAIR, DP-T2-weighted) and a whole-brain high resolution T1-weighted sequence obtained using a modified driven equilibrium Fourier transform (MDEFT) sequence (TE/TR = 2.4/7.92 ms, flip angle: 15°, voxel-size: 1 × 1 × 1 mm3) with a 3T Allegra MR imager (Siemens, Erlangen, Germany).

MRI-based quantification of cortical thickness was performed using the Freesurfer (v. 4.05) software package http://surfer.nmr.mgh.harvard.edu. This method has already been described in detail [16,17]. Images were first corrected for intensity of non-uniformity and registered via affine transformation (12 parameters) to Montreal Neurological Institute (MNI) space [18]. Then, images underwent a further intensity normalization using a different automated algorithm and were automatically skull stripped [16]. Next, the entire cortex was visually inspected prior to analysis. The data from 70 subjects were deemed to require manual correction, which included: a) setting intensity normalization control points where brain matter was erroneously skull stripped, b) adjusting watershed parameters of the skull strip, and c) visual inspecting and correcting of the automatic subcortical segmentation. All processes (i.e. skull stripping and segmentation) were inspected by an expert neuroradiologist who was blinded to the aim of the study.

For each subject, thickness measurements across the cortex were computed by finding the point on the gray-white matter boundary surface that was closest to a given point on the estimated pial surface (and vice versa) and obtaining the average of these two values [19]. The accuracy of the thickness measures derived from this technique was validated by direct comparisons with manual measurements on postmortem brains and direct comparisons with manual measurements on MRI data [20,21]. The surface representing the gray-white matter border was "inflated" [22]. Differences among individuals in the depth of gyri-sulci were normalized, and each subject's reconstructed brain was then morphed and registered to an average spherical surface representation that optimally aligned sulcal and gyral features across subjects [21]. Finally, cortical maps were smoothed with a 10-mm full-width at half maximum Gaussian kernel.

For each subject mean thickness values were then calculated for 33 regions of interest (ROIs) in each hemisphere, using the Destrieux atlas [23], implemented in the Freesurfer software.

### Statistical analyses

Association between TGF-β1 and cortical thickness was investigated using both a ROI-based and a vertex-based approach. The former was performed by calculating Pearson's *r *correlation coefficients between TGF-β1 serum levels and each ROI mean thickness. As this is a pilot study, we accepted the false-positive risk with an uncorrected level of significance set at *p *< .05. The vertex-based analysis was performed using the Qdec module, implemented in Freesurfer. For each hemisphere, estimation of statistical effects was generated by computing a general linear model (GLM) of the effects of TGF-β 1 on cortical thickness at each vertex. We modeled cortical thickness data using a linear regression analysis with TGF-β1 as the variable of interest and age as the nuisance variable. In this case, the threshold of statistical significance was increased at *p *< .001, which has been reported as a reasonable threshold for reporting results of neuroimaging studies when no corrections for multiple comparisons are made [e.g. [24]].

## Results and discussion

As shown in Table 1 ROI-based analyses revealed several brain areas (primarily in frontal and cingulate cortices of both hemispheres) in which there was a significant correlation between TGF-β1 serum values and cortical thickness. Specifically, correlations were found bilaterally in caudal anterior cingulate (r = .363, *p *= .0018 for left hemisphere; r = .330, *p *= .005 for right hemisphere) and bilaterally in the rostral anterior cingulate cortices (r = .247, *p *= .039 for left hemisphere; r = .439, *p *< .001 for right hemisphere); in the pars opercularis of the inferior frontal cortex (r = .273, *p *= .021) and in the rostral portion of the middle frontal area (r = .243, *p *= .043) and in the superior temporal cortex (r = .278, *p *= .019) of the right hemisphere only.

**Table 1:** Relationships between region of interest (ROI) mean cortical thicknesses and TGF-β1 serum levels for the 70 healthy participants

Left hemisphere ROIs			Right hemisphere ROIs		
	**Pearson's r**	**p-value**		**Pearson's r**	**p-value**

					
Caudal anterior cingulate	.363	.0018	Rostral anterior cingulate	.439	.0001
					
Rostral anterior cingulate	.247	.0393	Caudal anterior cingulate	.330	.0051
					
			Superior temporal	.278	.0194
					
			Inferior frontal opercular	.273	.0217
					
			Rostral middle frontal	.243	.0428

**Figure 1:**
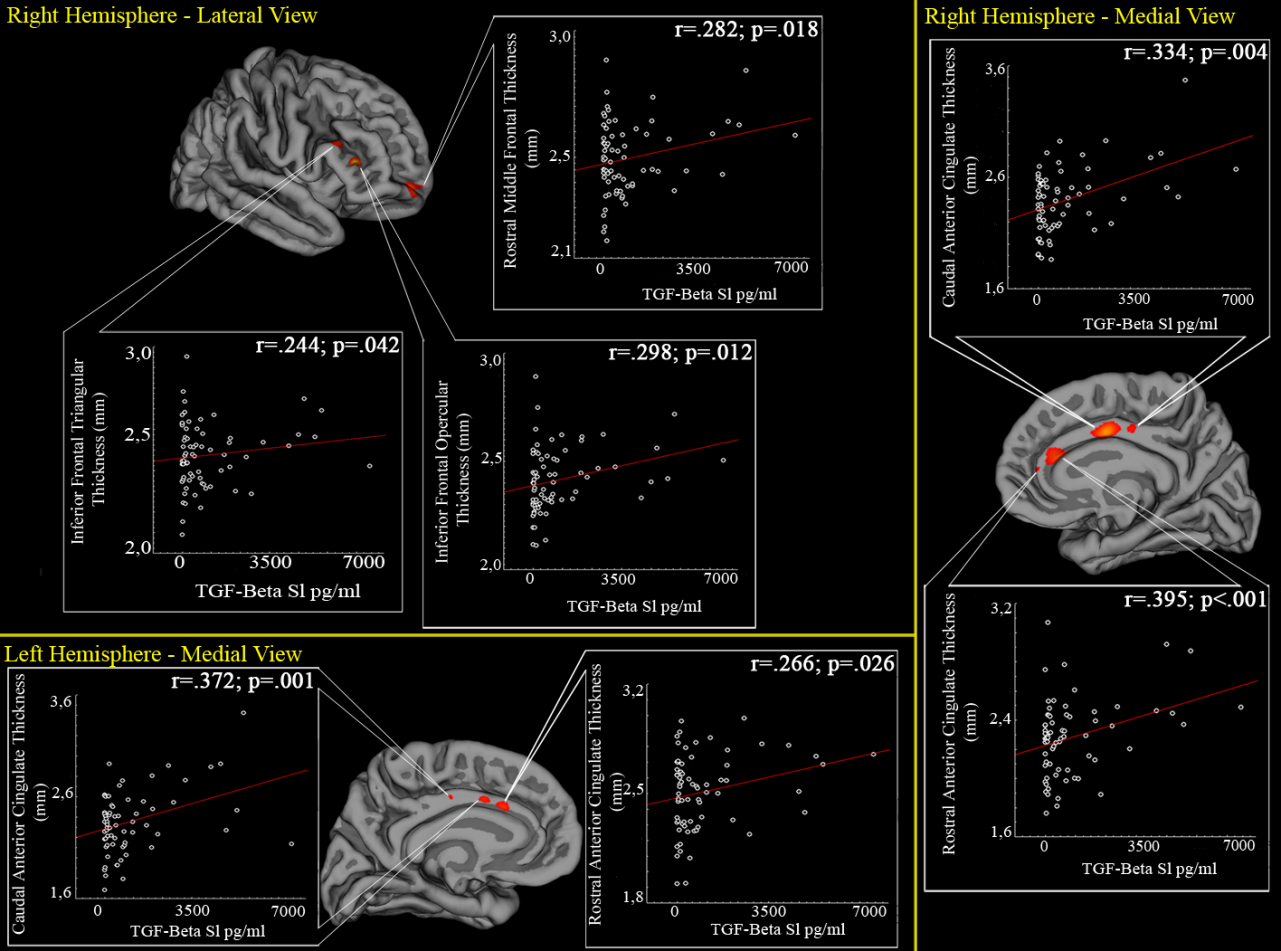
**Whole-brain vertex-wise analysis of correlation between cortical thickness and TGF-β1 serum levels**. Maps are superimposed on the pial cortical surface. Statistical results are reported in a -log p scale. For each subject, mean cortical thickness values of the clusters where significant results emerged were extracted. Scatterplots of cortical thickness and TGF-β1 levels are also reported, including Pearson's r coefficients and p values (linear fits are shown in red).

Results of the vertex-based analysis are summarized in Figure 1 and Table 2. In particular, significant results were found in rostral and caudal portions of bilateral anterior cingulate cortices, in the pars opercularis and triangularis of the right inferior frontal cortex and in the rostral part of the right middle frontal area.

**Table 2:** Vertex-based relationships between TGF-β1 and cortical thickness.

Left hemisphere				
**Anatomical region**	**Extent (mm^2^)**	**p**	**t**	**x,y,z {mm}**

Caudal anterior cingulate	40	.0005	3.29	-6, 22, 23
Rostral anterior cingulate	11	.0009	3.04	-1, 32, 7

**Right hemisphere**				

**Anatomical region**	**Extent (mm^2^)**	**p**	**t**	**x,y,z {mm}**

Inferior frontal lobe, pars triangularis	83	.00003	4.48	44, 33, 8
Rostral anterior cingulate	153	.0001	3.97	5, 29, -3
Caudal anterior cingulate	218	.00014	3.84	3, 25, 16
Rostral middle frontal	145	.0003	3.54	21, 58, -11
Inferior frontal lobe, pars opercularis	53	.0004	3.33	50, 25, 19

Coordinates are reported in MNI space

Thus, the results in this study, in which we analyzed TGF-β1 serum levels and brain cortical thickness in a large sample of healthy subjects, showed positive correlations in several brain areas, particularly the bilateral cingulate cortices and right frontal areas. Although these results are observational, they provide the first in-vivo support for the hypothesis that this cytokine has a putative protective effect in physiological conditions.

In this view, the role of TGF-β1 could be central in down-regulating inflammatory processes, because converging evidence suggests that peripheral levels of inflammation are associated with activation of central inflammatory mechanisms (through direct penetration of blood-brain barrier or via activation of the afferent vagus nerve) and might adversely affect cognition and brain structure. Indeed, Yaffe and colleagues [25] found that serum markers of inflammation, especially IL-6, likely predict cognitive decline in well-functioning elderly individuals. Along these lines, Marsland and coworkers [26] used a computational structural neuroimaging method (optimized voxel-based morphometry) to evaluate the relationship between plasma IL-6 levels and hippocampal grey matter volume in a sample of 76 relatively healthy community volunteers. They found a strong inverse correlation between IL-6 levels and hippocampal gray matter and argued that low-grade systemic inflammation might presage subclinical cognitive decline in part via structural neural pathways. This result has been confirmed in a more recent study [27] which showed a pattern of cortical thinning associated to levels of systemic inflammation in older persons without dementia.

Thus, we can speculate that if systemic inflammatory markers have a detrimental effect on the structural integrity of the brain (i.e. reduced cortical thickness), TGF-β1 might have a neuroprotective effect through its role in down-regulating inflammation.

Interestingly, the positive correlations between TGF-β1 levels and cortical thickness found in the present study were mainly located in brain areas involved in high-level cognitive processes (i.e. executive functions) such as the frontal areas and the cingulate cortex. Moreover, in animal studies the latter area was associated to a 37% increase of TGF-β1 mRNA 12 h after occlusion of the middle cerebral artery [28] and an increased expression of brain-derived neurotrophic factor (BDNF) 2 h after transient focal ischemia [29]. Therefore, the cingulate cortex might be a key area in which putative neuroprotective effects of TGF-β1occur, thus preventing negative agents such as aging, degeneration or cerebrovascular diseases. Nevertheless, the data presented here cannot completely address this issue because the study was cross-sectional and the participants were free from brain pathology.

A limitation of the present study is that we measured serum level of TGF-β1, which might not accurately reflect levels in the cerebrospinal fluid (CSF) or in brain regions. However, cytokines readily cross the blood-brain barrier, suggesting that serum levels should correlate well with levels in the CSF [30]. Further, there is evidence of a correlation between TGF-β levels in serum and CSF of patients with advanced AD [31].

## Conclusion

Overall, the results of the present study show that TGF-β1 serum concentrations are associated with greater cortical thickness in bilateral cingulate and right frontal areas in subjects without neuropsychiatric diseases. This suggests that TGF-β might also have a beneficial effect on the structural integrity of the brain even in physiological states.

Future studies should take on the challenge of longitudinally studying the role of TGF-β in protecting the brain from degeneration and injury, possibly by collecting data to analyze brain microstructural integrity (i.e. diffusion tensor imaging).

## Abbreviations

CSF: cerebrospinal fluid; IL-6: interleukin-6; MRI: magnetic resonance imaging; ROI: region of interest; TGF-β: transforming growth factor β.

## Competing interests

The authors declare that they have no competing interests.

## Authors' contributions

GS, CC and PB directed the work, contributed to designing the study and reviewed the data; FS performed ELISA assay and TGF-β measurements; FP performed MRI analyses and wrote the manuscript. All authors read and approved the final manuscript.

## References

[B1] Grainger DJ TGF-beta and atherosclerosis in man Cardiovasc Res 2007 74 213 222 10.1016/j.cardiores.2007.02.022 17382916

[B2] Gomes FC Sousa Vde O Romao L Emerging roles for TGF-beta1 in nervous system development Int J Dev Neurosci 2005 23 413 424 10.1016/j.ijdevneu.2005.04.001 15936920

[B3] Falk S Wurdak H Ittner LM Ille F Sumara G Schmid MT Draganova K Lang KS Paratore C Leveen P Suter U Karlsson S Born W Ricci R Gotz M Sommer L Brain area-specific effect of TGF-b signaling on Wnt-dependent neural stem cell expansion Cell Stem Cell 2008 2 472 483 10.1016/j.stem.2008.03.006 18462697

[B4] Makwana M Jones LJ Cuthill D Heuer H Bohatschek M Hristova M Friedrichsen S Ormsby I Bueringer D Koppius A Bauer K Doetschman T Raivich G Endogenous transforming growth factor beta 1. Suppresses inflammation and promotes survival in adult CNS J Neurosci 2007 27 1201 1213 10.1523/JNEUROSCI.2255-07.2007 PMC6673043 17942715

[B5] Shull MM Ormsby I Kier AB Pawlowski S Diebold RJ Yin M Allen R Sidman C Proetzel G Calvin D Targeted disruption of the mouse transforming growth factor-beta 1 gene results in multifocal inflammatory disease Nature 1992 359 693 699 10.1038/359693a0 1436033 PMC3889166

[B6] Bruno V Battaglia G Casabona G Copani A Caciagli F Nicoletti F Neuroprotection by glial metabotropic glutamate receptors is mediated by transforming growth factor-beta J Neursci 1998 18 9594 9600 10.1523/JNEUROSCI.18-23-09594.1998 PMC6793276 9822720

[B7] Tesseur I Wyss-Coray T A role for TGF-beta signaling in neurodegeneration: evidence from genetically engineered models Curr Alzheimer Res 2006 3 505 513 10.2174/156720506779025297 17168649

[B8] Lee HG Ueda M Zhu X Perry G Smith MA Ectopic expression of phospho-Smad2 in Alzheimer's disease: uncoupling of the transforming growth factor pathway? J Neurosci Res 2006 84 1856 1861 10.1002/jnr.21072 16998902

[B9] Town T Laouar Y Pittenger C Mori T Szekely CA Tan J Duman RS Flavell RA Blocking TGF-beta-Smad2/3 innate immune signaling mitigates Alzheimer-like pathology Nat Med 2008 14 681 687 18516051 10.1038/nm1781 PMC2649699

[B10] Mallat Z Gojova A Marchiol-Fournigault C Esposito B Kamaté C Merval R Fradelizi D Tedgui A Inhibition of transforming growth factor-beta signaling accelerates atherosclerosis and induces an unstable plaque phenotype in mice Circ Res 2001 89 930 934 10.1161/hh2201.099415 11701621

[B11] Gross CE Bednar MM Howard DB Sporn MB Transforming growth factor-beta 1 reduces infarct size after experimental cerebral ischemia in a rabbit model Stroke 1993 24 558 562 10.1161/01.STR.24.4.558 8465363

[B12] Henrich-Noack P Prehn JH Krieglstein J TGF-beta 1 protects hippocampal neurons against degeneration caused by transient global ischemia. Dose-response relationship and potential neuroprotective mechanisms Stroke 1996 27 1609 1614 10.1161/01.STR.27.9.1609 8784137

[B13] Folstein M Folstein S McHugh P Mini-mental state. A practical method for grading the cognitive state of patients for the clinician J Psychiatr Res 1975 12 189 198 10.1016/0022-3956(75)90026-6 1202204

[B14] Carlesimo G Caltagirone C Gainotti G The mental deterioration battery: normative data, diagnostic reliability and qualitative analyses of cognitive impairment. The group for the standardization of the mental deterioration battery Eur Neurol 1996 36 378 384 10.1159/000117297 8954307

[B15] McKhann G Drachman D Folstein M Katzman R Price D Stadlan E Clinical diagnosis of Alzheimer's disease: report of the NINCDS-ADRDA Work Group under the auspices of Department of Health and Human Services Task Force on Alzheimer's Disease Neurology 1984 34 939 944 6610841 10.1212/wnl.34.7.939

[B16] Dale AM Fischl B Sereno MI Cortical surface-based analysis. Part I: segmentation and surface reconstruction NeuroImage 1999 9 179 194 10.1006/nimg.1998.0395 9931268

[B17] Cerasa A Quattrone A Gioia MC Tarantino P Annesi G Assogna F Caltagirone C De Luca V Spalletta G Dysbindin C-A-T haplotype is associated with thicker medial orbitofrontal cortex in healthy population NeuroImage 2011 55 508 513 10.1016/j.neuroimage.2010.12.042 21184829

[B18] Collins DL Neelin P Peters TM Evans AC Automatic 3D intersubject registration of MR volumetric data in standardized Talairach space J Comput Assist Tomogr 1994 18 192 205 10.1097/00004728-199403000-00005 8126267

[B19] Fischl B Dale AM Measuring the thickness of the human cerebral cortex from magnetic resonance images Proc Natl Acad Sci USA 2000 97 11050 11055 10984517 10.1073/pnas.200033797 PMC27146

[B20] Rosas HD Liu AK Hersch S Glessner M Ferrante RJ Salat DH van der Kouwe A Jenkins BG Dale AM Fischl B Regional and progressive thinning of the cortical ribbon in Huntington's disease Neurology 2002 58 695 701 11889230 10.1212/wnl.58.5.695

[B21] Kuperberg GR Broome MR McGuire PK David AS Eddy M Ozawa F Goff D West WC Williams SC van der Kouwe AJ Salat DH Dale AM Fischl B Regionally localized thinning of the cerebral cortex in schizofrenia Arch Gen Psychiatry 2003 60 878 888 10.1001/archpsyc.60.9.878 12963669

[B22] Fischl B Sereno MI Dale AM Cortical surface-based analysis. II: inflation, flattening and a surface-based coordinate system NeuroImage 1999 9 195 207 10.1006/nimg.1998.0396 9931269

[B23] Destrieux C Fischl B Dale A Halgren E Automatic parcellation of human cortical gyri and sulci using standard anatomical nomenclature NeuroImage 2010 53 1 15 10.1016/j.neuroimage.2010.06.010 20547229 PMC2937159

[B24] Loring DW Meador KJ Allison JD Pillai JJ Lavin T Lee GP Balan A Dave V Now you see it, now you don't: statistical and methodological considerations in fMRI Epilepsy Behav 2002 3 539 547 10.1016/S1525-5050(02)00558-9 12609249

[B25] Yaffe K Lindquist K Penninx BW Simonsick EM Pahor M Kritchevsky S Launer L Kuller L Rubin S Harris T Inflammatory markers and cognition in well-functioning African-American and white elders Neurology 2003 61 76 80 12847160 10.1212/01.wnl.0000073620.42047.d7

[B26] Marsland AL Gianaros PJ Abramowitch SM Manuck SB Hariri AR Interleukin-6 covaries inversely with hippocampal grey matter volume in middle-aged adults Biol Psychiatry 2008 64 484 490 10.1016/j.biopsych.2008.04.016 18514163 PMC2562462

[B27] Fleischman DA Arfanakis K Kelly J Rajendran N Buchman AS Morris MC Barnes LL Bennett DA Regional cortical thinning and systemic inflammation in older persons without dementia J Am Ger Soc 2010 58 205 206 10.1111/j.1532-5415.2009.02657.x PMC2945260 20863359

[B28] Yamashita K Gerken U Vogel P Hossmann K Wiessner C Biphasic expression of TGF-beta1 mRNA in the rat brain following permanent occlusion of the middle cerebral artery Brain Res 1998 836 139 145 10415412 10.1016/s0006-8993(99)01626-1

[B29] Kokaia Z Zhao Q Kokaia M Elmér E Metsis M Smith ML Siesjö BK Lindvall O Regulation of brain-derived neurotrophic factor gene expression after transient middle cerebral artery occlusion with and without brain damage Exp Neurol 1995 136 73 88 10.1006/exnr.1995.1085 7589336

[B30] Banks WA Plotkin SR Kastin AJ Permeability of the blood-brain barrier to soluble cytokine receptors Neuroimmunomodulation 1995 2 161 165 10.1159/000096887 8646566

[B31] Chao CC Ala TA Hu S Crossley KB Sherman RE Peterson PK Frey WH II Serum cytokine levels in patients with Alzheimer's disease Clin Diagn Lab Immunol 1994 1 433 436 8556481 10.1128/cdli.1.4.433-436.1994 PMC368282

